# Emergency department routine data and the diagnosis of acute ischemic heart disease in patients with atypical chest pain

**DOI:** 10.1371/journal.pone.0241920

**Published:** 2020-11-05

**Authors:** Ki Hong Kim, Jeong Ho Park, Young Sun Ro, Ki Jeong Hong, Kyoung Jun Song, Sang Do Shin

**Affiliations:** 1 Department of Emergency Medicine, Seoul National University Hospital, Seoul, Korea; 2 Laboratory of Emergency Medical Services, Seoul National University Hospital Biomedical Research Institute, Seoul, Korea; 3 Department of Emergency Medicine, Seoul National University Boramae Medical Center, Seoul, Korea; University of Palermo, ITALY

## Abstract

**Background:**

Due to an aging population and the increasing proportion of patients with various comorbidities, the number of patients with acute ischemic heart disease (AIHD) who present to the emergency department (ED) with atypical chest pain is increasing. The aim of this study was to develop and validate a prediction model for AIHD in patients with atypical chest pain.

**Methods and results:**

A chest pain workup registry, ED administrative database, and clinical data warehouse database were analyzed and integrated by using nonidentifiable key factors to create a comprehensive clinical dataset in a single academic ED from 2014 to 2018. Demographic findings, vital signs, and routine laboratory test results were assessed for their ability to predict AIHD. An extreme gradient boosting (XGB) model was developed and evaluated, and its performance was compared to that of a single-variable model and logistic regression model. The area under the receiver operating characteristic curve (AUROC) was calculated to assess discrimination. A calibration plot and partial dependence plots were also used in the analyses. Overall, 4,978 patients were analyzed. Of the 3,833 patients in the training cohort, 453 (11.8%) had AIHD; of the 1,145 patients in the validation cohort, 166 (14.5%) had AIHD. XGB, troponin (single-variable), and logistic regression models showed similar discrimination power (AUROC [95% confidence interval]: XGB model, 0.75 [0.71–0.79]; troponin model, 0.73 [0.69–0.77]; logistic regression model, 0.73 [0.70–0.79]). Most patients were classified as non-AIHD; calibration was good in patients with a low predicted probability of AIHD in all prediction models. Unlike in the logistic regression model, a nonlinear relationship-like threshold and U-shaped relationship between variables and the probability of AIHD were revealed in the XGB model.

**Conclusion:**

We developed and validated an AIHD prediction model for patients with atypical chest pain by using an XGB model.

## Introduction

Acute ischemic heart disease (AIHD) is a major public health concern worldwide [1, 2]. One case of AIHD occurs approximately every 40 seconds in the United States [3]. Chest pain is the most common presenting complaint of AIHD [4, 5]. Various studies have shown that prompt identification of AIHD is important [6], and delayed treatment of AIHD leads to poor outcomes [7, 8]. However, making a precise and early diagnosis for chest pain is difficult [9–11]. Approximately 2% of patients with AIHD are missed on initial presentation to the emergency department (ED) [12].

Due to an aging population and the increasing proportion of patients with various comorbidities, the number of patients with AIHD who present to the ED with atypical chest pain is increasing [1, 13]. Although 12-lead electrocardiography (ECG) and cardiac biomarkers are key diagnostic tools for assessing AIHD [14–16] they may show nondiagnostic results in the initial ED period. High-sensitive cardiac troponin assays can also be used as diagnostic tests, but these assays have limitations, including variability and a lack of universal protocols to guide the reference interval [17, 18]. Several novel cardiac biomarkers, such as galectin-3 or heart-type fatty acid binding protein, have been investigated [19–21]. Approximately half of patients with AIHD have no diagnostic ECG findings, and only one-third of patients have increased cardiac biomarker levels at the time of ED presentation [22, 23]. In addition, because of the atypical presentation of symptoms, physicians may not order those tests in the early period of diagnosis. The likelihood of this happening may be increased when atypical chest pain is comorbid with various symptoms. In this scenario, AIHD may be missed, or its diagnosis may be delayed.

Demographic data, such as age and vital signs, are routinely collected during the initial examination, and they provide important information for screening for AIHD [5, 24]. Many patients may receive a complete blood count (CBC) and comprehensive metabolic panel (CMP) regardless of their characteristics when presenting in many EDs. In previous studies, components of the CBC were evaluated for their role in the pathogenesis of atherosclerosis and as risk predictors for ischemic heart disease (IHD) [25, 26]. Glucose, serum blood urea nitrogen, and creatinine have also been evaluated as indicators of metabolic disturbance in IHD progression [27]. However, the diagnostic performance of routinely collected information for patients with atypical chest pain has not been widely evaluated in previous studies.

Knowing the diagnostic performance of routinely collected data for the diagnosis of AIHD has clinical implications. First, such information can be used to screen for patients with possible AIHD among those who were not initially thought to have AIHD and therefore lack ECG and cardiac biomarker results. Second, because most of this information can be easily collected, it has many possible applications, especially for situations in which only limited testing can be performed. In low-level EDs or outpatient clinics, this information could be supportive and aid in screening patients with AIHD. Furthermore, this information could help inform health care providers’ decision making when requesting additional cardiac workups, such as serial ECG, cardiac biomarker follow-up, and coronary computed tomography angiography (CCTA).

Traditional models usually assume that each predictor is associated with outcomes in a linear fashion [28]. However, the association between routinely collected data and outcomes in atypical chest pain is uncertain. For example, components of routinely collected data, e.g., aspartate transaminase (GOT) and white blood cell count (WBC), may exhibit different patterns in relation to AIHD. There is also a possibility of high-order interaction between each predictor. A machine learning-based prediction model can be beneficial in this scenario because of its potential for use in evaluating complex data, including nonlinear data and high-order interactions [29].

The aim of this study was to develop and validate a machine learning-based prediction model for AIHD using routinely collected data (excluding 12-lead ECG and cardiac biomarker test results) in patients with atypical chest pain who visited the ED. We also evaluated the predictor variable importance of the model and how each predictor affected the probability of AIHD according to its value.

## Materials and methods

### Study design and ethical statements

This study was a single-center retrospective study in the ED of a large, urban, academic teaching hospital that receives ~60,000 ED visits annually.

This study was approved by the Institutional Review Board, and the requirement for informed consent was waived (IRB No. 1808-001-962). This study complied with the Declaration of Helsinki, and we adhered to the Transparent Reporting of a Multivariable Prediction Model for Individual Prognosis or Diagnosis statement on reporting predictive models [30].

### Study setting

The ED had approximately 10–12 emergency physicians, 9–11 emergency residents, 5 specialty board staff, 50–60 emergency nursing staff, and 15–17 emergency medical technicians from 2014 to 2018. A structured protocol for triage based on patients’ vital signs, brief history, and chief complaint was used. When patients visited the ED, dedicated ED nurses triaged patients based on a five-level scale (level 1: immediate, level 2: very urgent, level 3: urgent, level 4: less urgent, and level 5: not urgent) [31, 32]. Based on this scale, the most unstable patients, such as those with cardiac arrest or definite shock status, were classified as level 1. If a triage nurse found no evidence of severe shock or desaturation, all patients with chest pain were assigned to on-duty physicians in the cardiovascular section, which assists with efficient ED operation and sensitive diagnosis of cardiovascular disease. Patients with a chief complaint other than chest pain were included if they also had chest pain. After interviews and physical examinations, the primary physician completed the structured chest pain workup registry. They assigned all eligible patients into categories consisting of typical angina and other angina, as per the guidelines [33]. Typical angina was defined as having all three conditions: substernal squeezing chest pain, pain subsiding with rest or nitroglycerine administration, and pain aggravated by exercise [34, 35]. The registry was reviewed every other day by attending physicians for quality control of the management of patients with chest pain. If patients were suspected of having AIHD, consultation with a cardiology specialist was conducted for further management and admission.

### Selection of participants

We included adult (≥18 years old) nontrauma patients with chest pain who visited the ED from January 2014 to December 2018. Patients who presented with typical characteristics of angina were excluded because most needed to undergo further cardiac testing regardless of their characteristics. Patients who did not visit the ED until 1 week after symptom onset, received interhospital transfer, or presented with cardiac arrest or unstable vital signs and were classified as level 1 by a triage nurse, were excluded.

The study population was divided into a training cohort from which each of the machine learning prediction models was derived and a validation cohort in whom the prediction models were applied and tested. The training cohort was derived from data collected from January 2014 to December 2017; the validation cohort comprised data collected from January 2018 to December 2018.

### Data sources

We obtained data from three independent databases, including the ED administrative database, clinical data warehouse (CDW) database [36], and chest pain workup registry. Data were obtained from January 2014 to December 2018. The ED administrative database contains patients’ demographic characteristics, route of visit, time of visit, and diagnosis and disposition. The CDW database includes laboratory study results and imaging study results. The chest pain workup registry includes chest pain characteristics. We integrated the three databases using a common deidentified key to produce a comprehensive clinical dataset that contained sufficient information. If patients visited the ED multiple times within 7 days, only the data from the index visit were analyzed.

### Data description and preprocessing

Because our primary purpose of prediction was diagnosis of AIHD using routinely collected information aside from 12-lead ECG findings and cardiac biomarker measurements in the early ED period, only data available at the initial ED visit were used as prediction variables. For laboratory tests, we chose the initially retrieved CBC and CMP, which have been frequently used in most EDs. We selected 25 predictors according to eligibility, and a detailed description of the variables is presented in S1 Table. Among them, 23 predictors were continuous variables (age, vital signs, and blood laboratory test results), and there was a range of proportions of missing data (2.5% to 16.8%, S1 Table). Median imputation, which is a common method used to deal with missing values in machine learning models, was conducted [37]. Extreme value imputation was conducted for outlier replacement of continuous variables except for age. Using a training cohort, the 1^st^ percentile value of each continuous variable and 99^th^ percentile value of each continuous variable were defined as cutoff values. Values smaller than the 1^st^ percentile cutoff or larger than the 99^th^ percentile cutoff were defined as extreme values and replaced in both the training and validation cohorts. This method was used to develop a model that is less sensitive to extreme values, in order to reduce the effect of outliers [38].

### Outcome variable

The diagnosis of AIHD, which was extracted from the ED administrative database and CDW database, was used as the outcome. We defined patients as having AIHD if both of the following conditions were satisfied. First, among patients who visited the ED, the diagnostic code according to the International Statistical Classification of Diseases and Related Health Problems (ICD-10) needed to be between I-20 and I-25, which indicates IHD. The ED administrative database has two types of primary diagnostic codes: the final diagnostic codes at ED discharge and at hospital discharge. We defined the diagnostic code as positive for ischemic heart disease if a confirmative diagnostic code was found in any level of the discharge record. Next, a diagnosis of AIHD was accepted when coronary angiography (CAG) was performed during the patient’s hospital stay. We defined patients who were discharged without CAG results as nondiagnosed.

### Model development

We developed prediction models for the diagnosis of AIHD using extreme gradient boosting (XGB). The XGB algorithm operates by refitting a classifier iteratively to residuals of models [39]. The XGB algorithm was tuned using five-fold cross-validation. Grid and manual searches on the hyperparameters within the training cohort were conducted. The hyperparameters investigated in our models were as follows: nrounds: 50, 100, 150, 300, 350, 400, 450, and 500; max_depth: 2, 3, 4, 5, and 6; eta: 0.01, 0.02, 0.03, 0.04, 0.05, 0.1, and 0.3; gamma: 0, 0.05, 0.1. 0.5, 0.7, 0.9, and 1.0; colsample_bytree: 0.4, 0.6. 0.8, and 1.0; min_child_weight: 1, 2, and 3; and subsample: 0.5, 0.75, and 1.0. After tuning with cross-validations, we selected hyperparameters in the modeling as follows: nrounds: 450, max_depth = 2, eta = 0.02, gamma = 0.7, colsample_by_tree = 1, min_child_weight = 1, and subsample = 0.5.

### Statistical analysis

Sample size estimation was not conducted since this was designed as a hypothesis-generating epidemiological study, and all eligible patients were included to maximize the statistical power.

Characteristics including baseline characteristics, vital signs, laboratory test results, and the study outcome were compared between the training cohort and validation cohort using the t-test or Wilcoxon rank-sum test for continuous variables and the chi-square test or Fisher exact test for categorical variables, as appropriate [40]. Cardiac tests during hospital stays were also compared between the groups according to the study outcome.

A machine-learning model using the XGB algorithm was developed using 25 predictor variables. The XGB model has been widely used in the development of prediction models in the clinical field, and it has demonstrated good performance [41, 42]. The performance of the predictive model was evaluated by the area under the receiving operating characteristic curve (AUROC) as a primary measure. We assessed calibration power using the scaled Brier score, Hosmer-Lemeshow test, and a calibration plot in the validation cohort. The test characteristics of each model in the validation cohort, including the sensitivity, specificity, and positive and negative predictive values with 95% confidence intervals, were reported. The optimal cutoff probability for evaluation of the test characteristics was calculated using the Youden index. The variable importance of the XGB model was also reported. The XGB models were ranked by variable importance on the gain, which implies the relative contribution of the corresponding variable to the model calculated by taking each variable’s contribution for each tree in the model [43]. In addition, partial dependence plots were used to determine the marginal effect of features on the predicted outcome in the XGB model.

Two baseline models were developed to compare with the XGB model and traditional model. First, because troponin is a cardiac biomarker that is most commonly used in the diagnosis of AIHD, a single-variable logistic regression model using troponin was developed to assess the usability of the XGB model in the clinical setting. Second, to compare the performance of the machine learning model and the traditional model, a logistic regression model of all predictors was developed. The variable importance of the logistic regression model was also reported. The logistic regression model was ranked by variable importance using z-statistics (the beta estimate divided by the standard error of beta). Partial dependence plots for the logistic regression model were also evaluated. Comparison of the AUROC between the XGB model and the two baseline models was performed using the De-Long test [44].

A p-value of 0.05 was considered statistically significant. All analyses were performed using R, version 3.5 (R Foundation for Statistical Computing, Vienna, Austria) with packages including caret and xgboost for the analysis of the machine learning algorithms.

## Results

### Characteristics of study subjects

**Fig 1:**
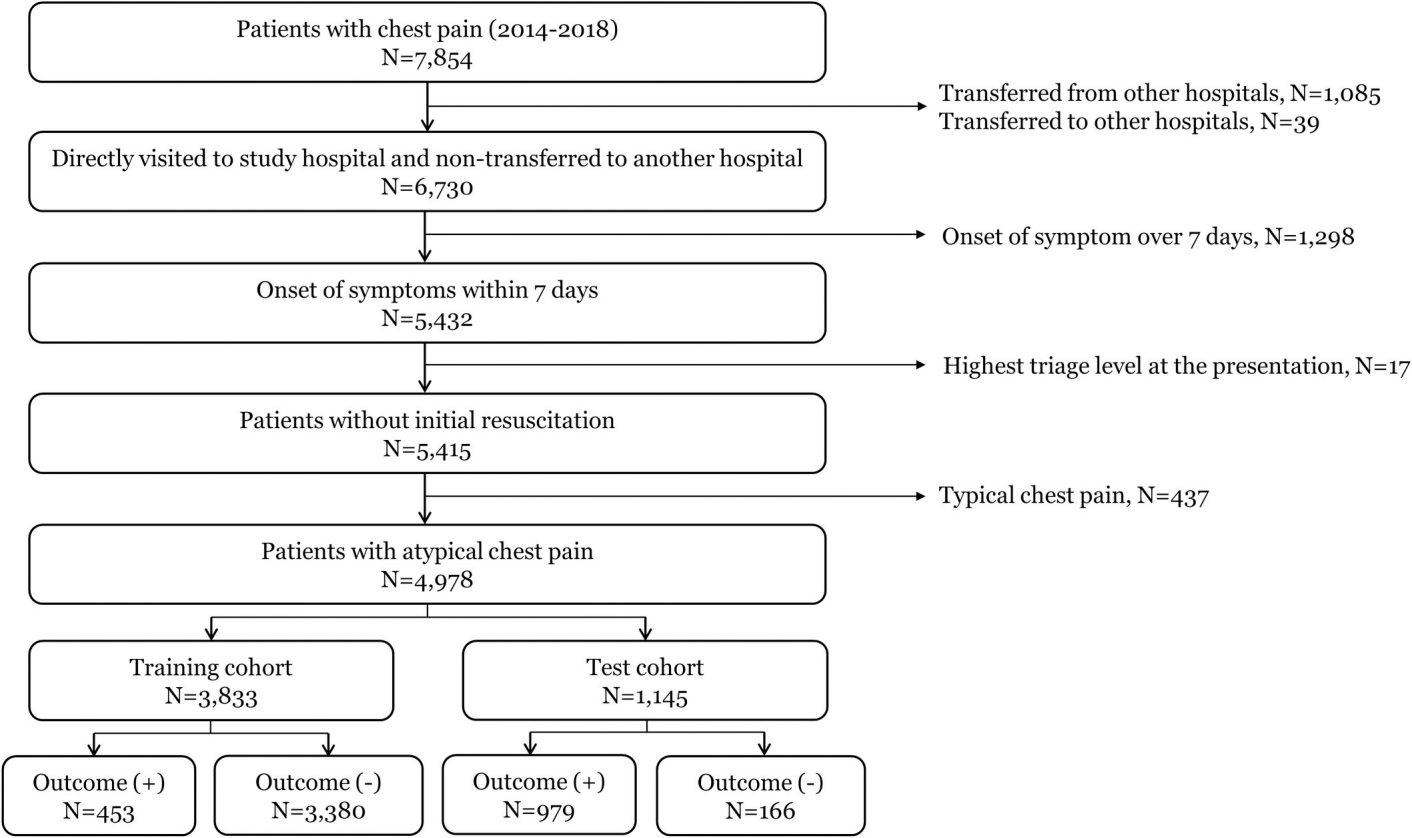
Patient flow chart.

Altogether, 10,217 patients were screened from the comprehensive dataset. After excluding patients who were transferred from other hospitals (N = 1,085), transferred to another hospital (N = 39), had symptom onset more than 7 days before their ED visit (N = 1,298), or had the highest triage level at presentation (N = 17), 5,415 patients remained. Among them, 437 patients with typical chest pain were excluded; therefore, 4,978 patients were included in the final analysis (Fig 1). The distribution of chest pain characteristics and proportion of patients with AIHD among the 5,415 patients are presented in S1 Fig.

Among the 4,978 patients, 619 (12.4%) patients were diagnosed with AIHD. The study population was divided into 2 cohorts: a training cohort of 3,833 patients and a validation cohort of 1,145 patients. The baseline characteristics of the training cohort and validation cohort are shown in Table 1. Compared to patients in the training cohort, those in the validation cohort were more likely to visit the ED earlier, to call emergency medical services (EMS), to have a higher blood pressure and heart rate, and to be diagnosed as having AIHD (11.8% vs. 14.5%). Several laboratory test results, such as WBC, total protein level, albumin level, and electrolyte level, were significantly different between the two groups, and mortality was low in both the training and validation groups (0.5% and 0.1%, respectively; p = 0.071) (Table 1).

**Table 1:** Comparison of participant characteristics in the development and validation cohorts.

		No. (%) or Median (IQR)			
		Original cohort	Training cohort	Validation cohort	*p*-value
Variables		(N = 4,978)	(N = 3,833)	(N = 1,145)	
Baseline characteristics					
	Age, mean (SD), years	60.5 (16.6)	60.3 (16.6)	60.8 (16.5)	0.355
	Male sex	2,701 (54.3%)	2,087 (54.4%)	614 (53.6%)	0.648
	Onset to ED visit time, hours	7.5 (1.6–46.9)	7.8 (1.6–48.0)	6.5 (1.6–27.2)	0.014
	EMS use	1,258 (25.3%)	936 (24.4%)	322 (28.1%)	0.013
Vital signs					
	Systolic blood pressure, mmHg	146 (130.0–171.0)	145 (129.0–169.0)	152 (134.0–175.0)	<0.001
	Diastolic blood pressure, mmHg	83 (74.0–94.0)	83 (73.0–93.0)	84 (75.0–94.0)	0.001
	Heart rate, beats per min	79 (69.0–91.0)	79 (69.0–92.0)	78 (68.0–90.0)	0.022
	Respiratory rate, breaths per min	18 (17.0–20.0)	18 (18.0–20.0)	18 (16.0–20.0)	<0.001
	Body temperature, °C	36.4 (36.2–36.6)	36.4 (36.2–36.6)	36.4 (36.2–36.5)	0.977
Laboratory data					
	White blood cell count, ×10^3^/μl	7 (5.7–8.8)	7.1 (5.7–8.9)	6.8 (5.5–8.5)	<0.001
	Hemoglobin level, g/dl	13.4 (12.1–14.7)	13.4 (12.1–14.7)	13.3 (11.9–14.5)	0.003
	Platelet count, ×10^3^/μl	224 (183.0–268.0)	225 (185.0–269.0)	220 (181.0–265.0)	0.019
	Total bilirubin level, mg/dL	0.6 (0.5–0.9)	0.6 (0.5–0.9)	0.6 (0.5–0.8)	0.02
	Serum aspartate transaminase level, IU/L	22 (18.0–29.0)	22 (18.0–29.0)	22 (18.0–29.0)	0.966
	Serum alanine aminotransferase level, IU/L	20 (14.0–29.0)	20 (14.0–29.0)	20 (14.0–29.0)	0.533
	Serum alkaline phosphatase level, IU/L	64 (52.0–79.0)	64 (53.0–79.0)	63 (51.0–78.0)	0.093
	Total serum protein level, g/dL	7.1 (6.7–7.5)	7.2 (6.8–7.5)	6.8 (6.5–7.2)	<0.001
	Serum albumin level, g/dL	4.2 (3.9–4.4)	4.2 (3.9–4.4)	4 (3.8–4.3)	<0.001
	Blood urea nitrogen level, mg/dl	15 (12.0–19.0)	15 (12.0–19.0)	15 (12.0–19.0)	0.671
	Serum creatinine level, mg/dL	0.9 (0.7–1.1)	0.9 (0.7–1.1)	0.9 (0.7–1.0)	0.486
	Serum sodium level, mmol/L	140 (138.0–142.0)	140 (138.0–142.0)	140 (138.0–142.0)	0.850
	Serum potassium level, mmol/L	4.2 (3.9–4.5)	4.2 (3.9–4.5)	4.1 (3.9–4.5)	<0.001
	Serum chloride level, mmol/L	104 (102.0–106.0)	104 (102.0–106.0)	104 (102.0–106.0)	0.001
	Carbon dioxide level, Total, mmol/L	24.2 (3.1)	24 (3.0)	25.1 (3.6)	<0.001
	Serum calcium level, mg/dL	9.1 (8.7–9.5)	9.2 (8.8–9.5)	9 (8.6–9.3)	<0.001
	Serum glucose level, mg/dL	115 (101.0–141.0)	115 (102.0–142.0)	113 (101.0–141.0)	0.103
Outcome					
	AIHD, no. (%)	619 (12.4%)	453 (11.8%)	166 (14.5%)	0.018
	In-hospital mortality, no. (%)	22 (0.4%)	21 (0.5%)	1 (0.1%)	0.071

IQR, interquartile range; ED, emergency department; EMS, emergency medical service; AIHD, acute ischemic heart disease; no., number; SD, standard deviation.

All continuous variables were preprocessed for extreme values by replacing 1st and 99^th^ percentiles.

### Main results

The characteristics of the study patients according to the study outcome are presented in Table 2.

**Table 2:** Comparison of participants’ characteristics according to study outcome.

		No. (%) or Median (IQR)			
		Original cohort	Non-AIHD	AIHD	*p*-value
Variables		(N = 4,978)	(N = 4,359)	(N = 619)	
Baseline characteristics					
	Age, mean (SD), years	60.5 (16.6)	59.5 (17.0)	67.2 (11.5)	<0.001
	Male sex	2,701 (54.3%)	2,264 (51.9%)	437 (70.6%)	<0.001
	Onset to ED visit time, hours	7.5 (1.6–46.9)	7.7 (1.7–46.6)	5.3 (1.4–47.7)	0.118
	EMS use	1,258 (25.3%)	1,063 (24.4%)	195 (31.5%)	<0.001
Vital signs					
	Systolic blood pressure, mmHg	146 (130.0–171.0)	146 (130.0–170.0)	151 (131.0–177.5)	0.009
	Diastolic blood pressure, mmHg	83 (74.0–94.0)	83 (74.0–93.0)	84 (73.0–94.0)	0.363
	Heart rate, beats per min	79 (69.0–91.0)	79 (69.0–92.0)	76 (66.0–87.0)	<0.001
	Respiratory rate, breaths per min	18 (17.0–20.0)	18 (17.0–20.0)	18 (18.0–20.0)	0.703
	Body temperature, °C	36.4 (36.2–36.6)	36.4 (36.2–36.6)	36.4 (36.2–36.5)	<0.001
Laboratory data					
	White blood cell count, ×10^3^/μl	7 (5.7–8.8)	6.9 (5.6–8.7)	7.8 (6.2–9.8)	<0.001
	Hemoglobin level, g/dl	13.4 (12.1–14.7)	13.3 (12.1–14.6)	13.9 (12.3–15.0)	<0.001
	Platelet count, ×10^3^/μl	224 (183.0–268.0)	225 (184.0–270.0)	217 (179.0–259.5)	0.012
	Total bilirubin level, mg/dL	0.6 (0.5–0.9)	0.6 (0.5–0.9)	0.6 (0.5–0.8)	0.911
	Serum aspartate transaminase level, IU/L	22 (18.0–29.0)	22 (18.0–28.0)	25 (19.0–34.0)	<0.001
	Serum alanine aminotransferase level, IU/L	20 (14.0–29.0)	19 (14.0–29.0)	22 (15.0–32.0)	<0.001
	Serum alkaline phosphatase level, IU/L	64 (52.0–79.0)	64 (52.0–78.0)	66 (54.0–80.0)	0.121
	Total serum protein level, g/dL	7.1 (6.7–7.5)	7.1 (6.7–7.5)	7 (6.7–7.4)	0.003
	Serum albumin level, g/dL	4.2 (3.9–4.4)	4.2 (3.9–4.4)	4.1 (3.9–4.3)	<0.001
	Blood urea nitrogen level, mg/dl	15 (12.0–19.0)	15 (12.0–19.0)	16 (13.0–20.0)	<0.001
	Serum creatinine level, mg/dL	0.9 (0.7–1.1)	0.9 (0.7–1.0)	1 (0.8–1.1)	<0.001
	Serum sodium level, mmol/L	140 (138.0–142.0)	140 (138.0–142.0)	140 (138.0–141.0)	0.355
	Serum potassium level, mmol/L	4.2 (3.9–4.5)	4.2 (3.9–4.5)	4.2 (3.9–4.5)	0.022
	Serum chloride level, mmol/L	104 (102.0–106.0)	104 (102.0–106.0)	104 (102.0–106.0)	0.219
	Total carbon dioxide level, mmol/L	24.2 (3.1)	24.2 (3.1)	23.9 (3.1)	0.08
	Serum calcium level, mg/dL	9.1 (8.7–9.5)	9.1 (8.7–9.5)	9.1 (8.7–9.4)	0.12
	Serum glucose level, mg/dL	115 (101.0–141.0)	113 (101.0–139.0)	129 (109.0–171.0)	<0.001
Outcome					
	In-hospital mortality, no. (%)	22 (0.4%)	13 (0.3%)	9 (1.5%)	0.001

IQR, interquartile range; AIHD, acute ischemic heart disease; ED, emergency department; EMS, emergency medical service; no., number; SD, standard deviation.

All continuous variables were preprocessed for extreme values by replacing 1st and 99^th^ percentiles.

Compared to patients without AIHD, those with AIHD were more likely to be older men, to call EMS, and to have a higher systolic blood pressure (SBP) and lower heart rate. Among the 17 laboratory test results, 11 were significantly different between patients with and without AIHD (Table 3).

**Table 3:** Comparison of participants’ cardiac workup results according to the study outcome.

	No. (%) or Median (IQR)			
	Original cohort	Non-AIHD	AIHD	*p*-value
Variables	(N = 4,978)	(N = 4,359)	(N = 619)	
12-lead ECG conducted in the ED	4,926 (99.0%)	4,309 (98.9%)	617 (99.7%)	0.094
Troponin I administered in the ED	4,833 (97.1%)	4,223 (96.9%)	610 (98.5%)	0.029
Troponin I, ng/ml	0.01 (0.01–0.01)	0.01 (0.01–0.01)	0.03 (0.01–0.22)	<0.001
Troponin I ≥0.03, ng/ml	832 (17.2%)	520 (12.3%)	312 (51.1%)	<0.001
CCTA conducted	1,002 (20.1%)	928 (21.3%)	74 (12.0%)	<0.001
CAG conducted	636 (12.8%)	17 (0.4%)	619 (100.0%)	<0.001
PCI conducted	367 (7.4%)	1 (<0.1%)	366 (59.1%)	<0.001

IQR, interquartile range; AIHD, acute ischemic heart disease; ED, emergency department; CCTA, coronary computed tomography angiography; CAG, coronary angiography; PCI, percutaneous coronary intervention; ECG, electrocardiography.

Among the 4,978 patients, most underwent 12-lead ECG (99.0%) and cardiac biomarker analysis (97.1%). Troponin I was elevated in 51.1% of AIHD patients and 12.3% of non-AIHD patients (p < 0.001). CCTA was conducted in 21.3% of non-AIHD patients and 12.0% of AIHD patients (p < 0.001). Seventeen non-AIHD patients underwent CAG, and their main diagnoses were as follows: heart failure, 2; arrhythmia, 2; pericardial disease, 2; cardiomyopathy, 2; and other, 2.

**Fig 2:**
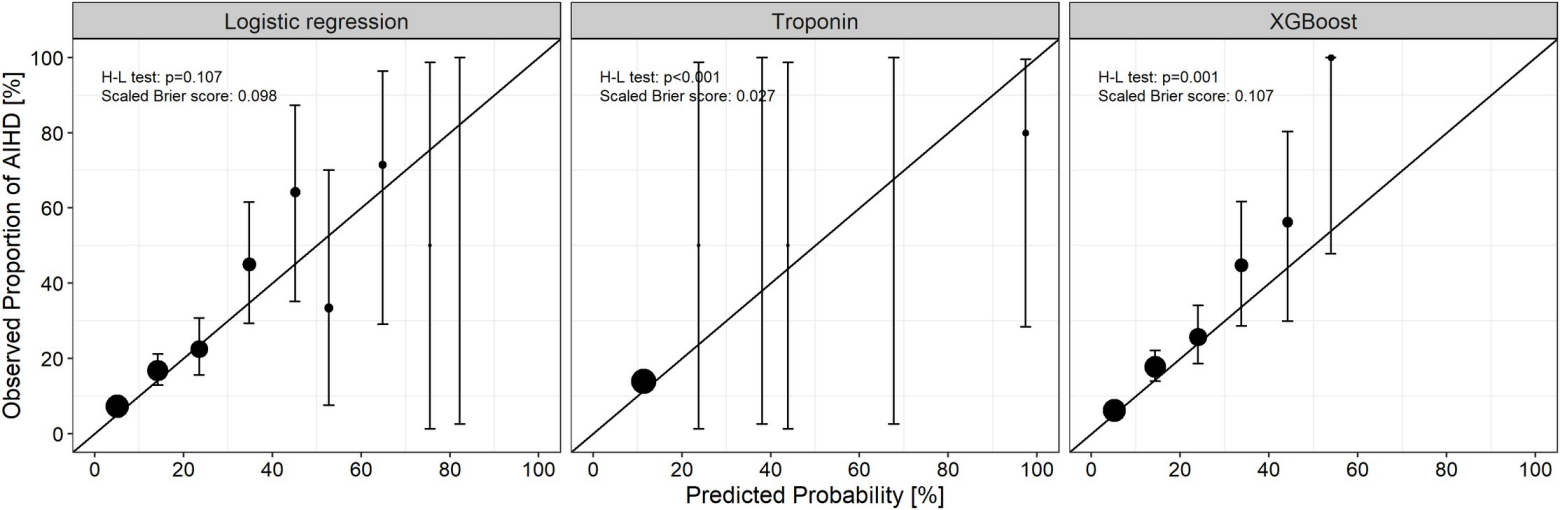
Calibration plots for acute ischemic heart disease in the validation cohort. The observed probability of acute ischemic heart disease (with a 95% confidence interval) is plotted against predicted good neurological recovery by 10% intervals of the predicted probability. Point size indicates the relative number of observations in a group. AIHD, acute ischemic heart disease.

Classification results of the machine learning models in the validation cohort are presented in Table 4. There was no significant difference in AUROC between the XGB model and the baseline models (Table 4). The test characteristics of the prediction models are also shown in Table 4. The accuracy and F1 scores of the logistic regression model and XGB model were similar (logistic regression model: 0.66 and 0.36; XGB model: 0.67 and 0.36, respectively). Calibration metrics are presented in Fig 2. Calibration was poor in patients with a high predicted probability of AIHD in all prediction models.

**Table 4:** Discrimination and test characteristics of acute ischemic heart disease prediction models in the validation cohort.

Model tested	AUROC(95% CI)	Cutoff probability*	TP	FN	TN	FP	Accuracy	Sensitivity	Specificity	PPV	NPV	F1Score
Troponin model	0.73 (0.69–0.77)	0.112	85	81	894	85	0.86	0.51	0.91	0.50	0.92	0.51
LR model	0.73 (0.70–0.77)	0.119	111	55	646	333	0.66	0.67	0.66	0.25	0.92	0.36
XGB model ^†^	0.75 (0.71–0.79)	0.127	107	59	659	320	0.67	0.64	0.67	0.25	0.92	0.36

*Cutoff was calculated using the Youden index.

^†^*p*-value for comparison of AUROC with baseline models: 0.600 for the troponin model and 0.277 for the logistic regression model.

AUROC, area under the receiver operating characteristic curve; 95% CI, 95% confidence interval; TP, true positive; FN, false negative; TN, true negative; FP, false positive; PPV, positive predictive value; NPV, negative predictive value; LR, logistic regression; XGB, extreme gradient boosting.

### Variable importance and partial dependence plot

**Fig 3:**
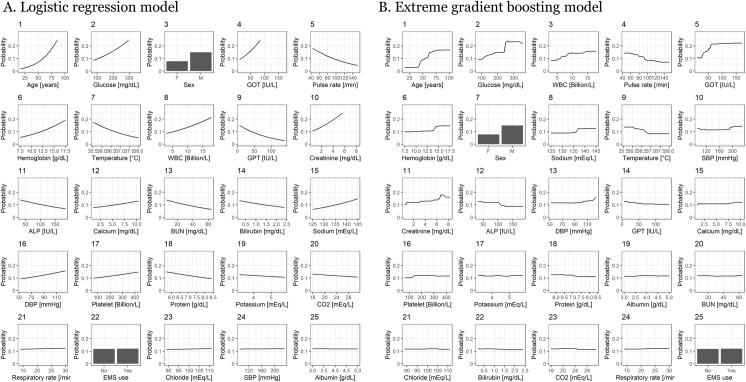
A. Partial dependence plot for the A) logistic regression model and B) extreme gradient boosting model. The orders of each plot are according to the variable importance of each model. GOT, aspartate transaminase; WBC, white blood cell count; GPT, alanine aminotransferase; ALP, alkaline phosphatase; BUN, blood urea nitrogen; DBP, diastolic blood pressure; CO2, carbon dioxide; SBP, systolic blood pressure; EMS, emergency medical service.

Variable importance was calculated for the logistic regression model and XGB model (S2 Table). Age and glucose ranked first and second in both models, and there was variability in variable importance between the models. The relationship between the probability of AIHD and all features in the models was demonstrated according to importance, as shown in Fig 3. A notable nonlinear trend was observed in several features in the XGB model (Fig 3).

## Discussion

We applied a machine-learning algorithm to patients with atypical chest pain who visited the ED in order to generate a predictive model of AIHD. We found that routinely collected data showed considerable predictive power, comparable to that of cardiac biomarkers. We also found that there were differences in variable importance between the XGB model and logistic regression model. Unlike the logistic regression model, many predictors showed nonlinear associations with the study outcome in the XGB model (Fig 3).

We found that discrimination power was comparable between the XGB model and troponin model. Cardiac biomarkers are important for the diagnosis of AIHD in patients with chest pain. Our findings suggest that a machine learning model with a combination of less relevant predictors could achieve equivalent performance to biomarkers with biological relevance. Because the XGB model showed good calibration in patients with a low probability of AIHD and identified most patients as having a low probability of AIHD (Fig 2), it can be helpful when deciding whether to conduct further cardiac tests, such as CCTA, in patients with a low risk of AIHD even when ECG and troponin findings are not available. In our study, one-fifth of non-AIHD patients underwent CCTA (Table 3). Decreasing the proportion of patients receiving CCTA may reduce radiological hazards and length of stay for those patients and preserve ED resources [45].

We also found that the discrimination power was comparable between our model and the logistic regression model. However, we found that the variable importance was markedly different between the XGB model and logistic regression model (S2 Table). The inherent linear relationship between a feature and the outcome of AIHD could contribute to different variable importance between the two models. SBP was the 24^th^ most important variable in the logistic regression model but the 9^th^ most important variable in the XGB model. Because the relationship between SBP and AIHD was U-shaped in the XGB model, the logistic regression model could not detect an important relationship between SBP and AIHD. A U-shaped relationship between SBP and the outcome of AIHD was also reported in a previous study [46]. GOT was among the top 5 most important variables in both models. GOT was proportionate to the risk of AIHD in the logistic regression model, but in the XGB model, that risk did not increase for GOT >75 IU/L. GOT is one of the oldest known biomarkers for AIHD [47]. However, because GOT originates from skeletal muscles or the liver rather than the heart, a high GOT level usually reflects diseases in those organs and not AIHD [48]. Therefore, the nonlinear relationship derived from the XGB model may be more compatible with biological relevance. A similar finding was also shown for glucose and WBC. Hyperglycemia and leukocytosis were associated with a high risk of AIHD in previous studies [49, 50]. However, severe hyperglycemia and leukocytosis are usually associated with an endocrine problem or an infection acquired in the ED, respectively.

The similarity of discrimination power between the XGB model and logistic regression model may be due to the preprocessing method used in our study. We replaced the extreme values of each feature in preprocessing to develop a less sensitive prediction model for outliers [38]. Because of the linear relationship between the predictor and the outcome in the logistic regression model, patients with extreme values of predictors, such as glucose, WBC, or GOT, may be classified as having AIHD in the logistic regression model. This result could diminish the discrimination power of the logistic regression model because those laboratory results could be caused by other diseases, such as an endocrine problem, infection, or hepatitis, rather than AIHD. Additionally, the inclusion of all predictors in both the XGB and logistic regression models also contributed to the similar predictive power between the models. Because the variable importance and internal processing of predictors are different between the two models, the performance of each model may be different when limited information can be used in constructing it.

We evaluated the predictive performance of routinely collected information for AIHD in patients with atypical chest pain. Atypical chest pain has been reported to have a high prevalence in AIHD patients, especially in the elderly and in women [51–53]. Because the diagnosis of AIHD in atypical chest pain is often challenging, decision support tools for the accurate diagnosis of these patients could result in decreased misdiagnosis, inappropriate discharge, and in-hospital mortality [51]. As chest pain characteristics are not routinely collected data in many administrative databases or CDW, this group of patients is not easily identified. In this study, merging various databases allowed us an opportunity to evaluate hypotheses that could not solely be addressed by one database. We found that chest pain characteristics critically affected the probability of AIHD. Patients with three typical characteristics showed a 9.5 times higher probability of AIHD compared to patients without typical characteristics. Even in patients with one typical characteristic, the probability of AIHD doubled compared to patients without typical characteristics (S1 Fig). Because uncertainty in AIHD diagnosis increases with lower numbers of typical characteristics, the utility of prediction models is greater in patients with few characteristics.

ECG and serum cardiac biomarkers are well-known predictors for diagnosing AIHD [54, 55]. Both variables can achieve a high level of performance, and prediction models and stratification tools tend to utilize these variables [10, 56]. However, we did not use these variables because we considered that each variable would show dominant significant predictive power in the model by itself, which would make it difficult to evaluate other clinically important variables. Moreover, we expected that prediction models for AIHD that do not utilize those test results would have their own clinical implications. Our model can be applied in various settings without high-level laboratory facilities or specialists who interpret ECG findings. Even in facilities with many resources, nurses can undertriage, physicians may overlook diagnoses, or ED overcrowding can create issues [57, 58]. Our model could also be applied to patients who visit the ED after their routine laboratory test was performed in an outpatient clinic or another hospital. Because our model can be applied in situations where some laboratory findings are missing, the potential coverage of our model is extensive.

This study has several limitations. First, the final diagnosis of the patient was defined based on the diagnostic code and procedure result recorded in his/her electronic medical record. This definition did not include whether the culprit lesion was observed or whether further intervention, such as ballooning or stenting, was performed. We focused on CAG rather than percutaneous coronary intervention (PCI) since this population should not be overlooked in the ED, even if they do not ultimately undergo PCI. Second, this study was conducted based on a population that visited one tertiary ED; thus, further external validation is required for data generalization. Third, only patients with chest pain who visited the ED were enrolled, and the chief complaint was determined by a triage nurse. Patients with dyspnea, syncope, or palpitation but no chest pain at the time of their ED visit were not included. In addition, patients with altered mental state who could not verbalize their complaint were not enrolled.

## Conclusion

In summary, we used the XGB algorithm to develop and validate prediction models for AIHD in patients with atypical chest pain who visited the ED. Our prediction model showed similar performance to the troponin and logistic regression models for detecting AIHD. However, we identified a notable nonlinear relationship between predictors and the study outcome and a different variable importance pattern by using the XGB model. Further prospective validation of our results is warranted, and a response protocol based on our model should be evaluated. Because we developed our prediction model using routinely collected data, a rapid response system based on our model may be applied more broadly to critical patients in the emergency setting. An automatic screening process that uses basic important variables and routine testing should be considered.

## Supporting information

S1 Fig Number of patients with certain chest pain characteristics and proportion of patients with acute ischemic heart disease in each population. AIHD, acute ischemic heart disease.
(DOCX)

S1 Table List of analyzed variables. *Values of median, 1st percentile and 99th percentile of continuous variables were extracted from the training cohort. All continuous variables were preprocessed for extreme values by replacing the 1st and 99th percentiles. ED, emergency department; N/A, not applicable; CBC, complete blood count.
(DOCX)

S2 Table Full ranking of important variables for the prediction models. Variable importance was determined based on the z value (logistic regression) and gain (XGB) obtained from the training cohort. XGB, extreme gradient boosting; EMS, emergency medical service.
(DOCX)

S1 Data (ZIP)
