# Supplementary material for: Emergency department routine data and the diagnosis of acute ischemic heart disease in patients with atypical chest pain
Source: PLoS One. 2020 Nov 5;15(11):e0241920. doi: 10.1371/journal.pone.0241920 (PMC7644067; doi:10.1371/journal.pone.0241920)

**S1 Fig. Number of patients with certain chest pain characteristics and proportion of patients with acute ischemic heart disease in each population.** AIHD, acute ischemic heart disease.


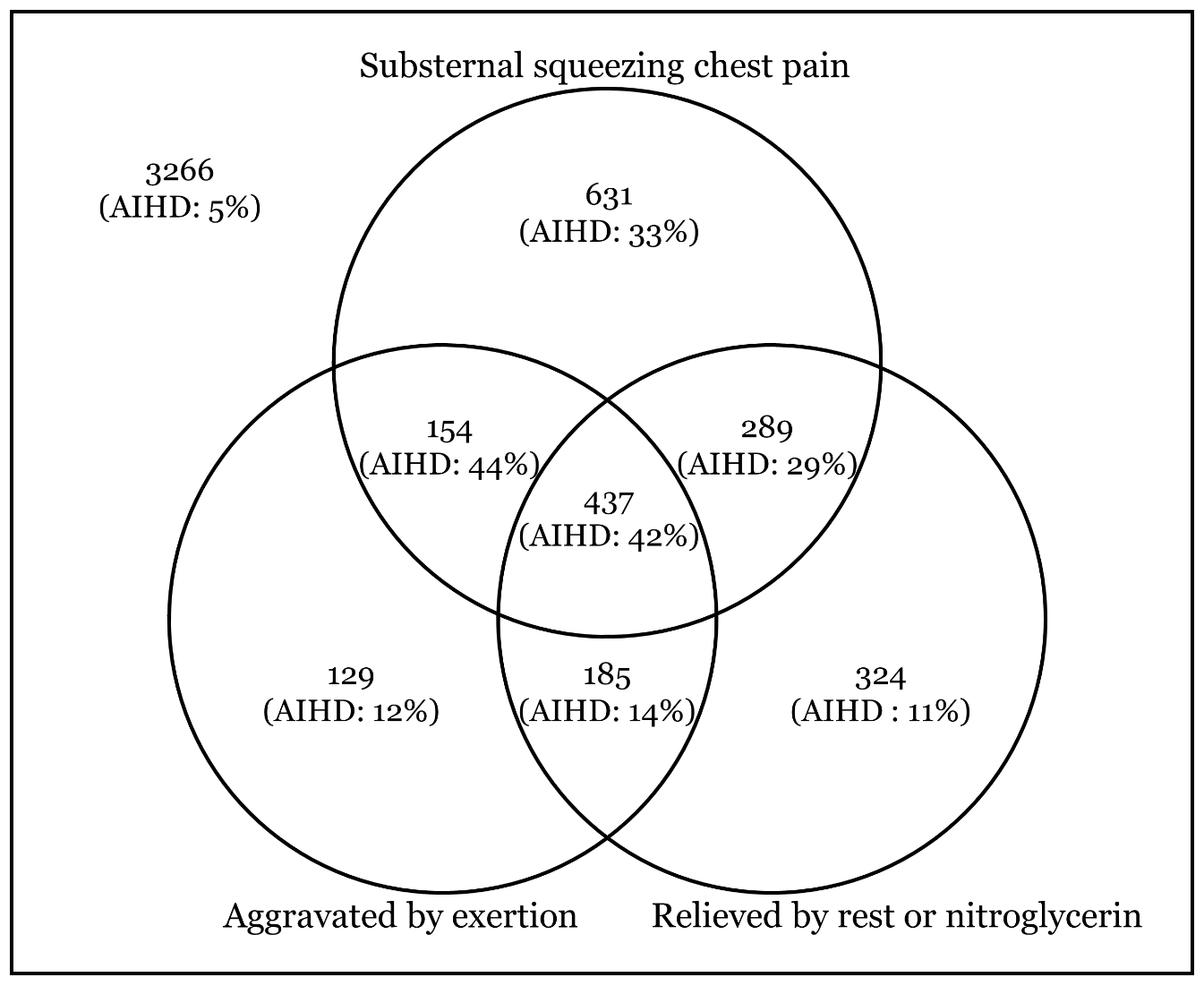

Supplement: S1 Fig — AIHD, acute ischemic heart disease. (DOCX) [file pone.0241920.s001.docx]
