# Supplementary material for: Emergency department routine data and the diagnosis of acute ischemic heart disease in patients with atypical chest pain
Source: PLoS One. 2020 Nov 5;15(11):e0241920. doi: 10.1371/journal.pone.0241920 (PMC7644067; doi:10.1371/journal.pone.0241920)
Supplement: S1 Table — *Values of median, 1st percentile and 99th percentile of continuous variables were extracted from the training cohort. All continuous variables were preprocessed for extreme values by replacing the 1st and 99th percentiles. ED, emergency department; N/A, not applicable; CBC, complete blood count. (DOCX) [file pone.0241920.s002.docx]

**S1 Table. List of analyzed variables.**

| Variable name | Description | Value | Type of raw data | Missing n (%) | Median^*^ | 1st percentile^*^ | 99th percentile^*^ |
| --- | --- | --- | --- | --- | --- | --- | --- |
| age | Patients’ age | years, at the time of ED visit | Continuous | 0 (0) | 62 | 20 | 89 |
| sex | Patients’ sex | Male=1, Female=0 | Binary | 0 (0) | N/A | | |
| ems | Call public emergency medical system to visit ED | Yes=1, No=0 | Binary | 0 (0) | N/A | | |
| sbp | Systolic blood pressure | mmHg, at the time of ED visit | Continuous | 0 (0) | 145 | 92 | 230 |
| dbp | Diastolic blood pressure | mmHg, at the time of ED visit | Continuous | 0 (0) | 83 | 52 | 124 |
| pr | Heart rate | beats per minute, at the time of ED visit | Continuous | 0 (0) | 79 | 44 | 152 |
| rr | Respiratory rate | breaths per minute, at the time of ED visit | Continuous | 0 (0) | 18 | 14 | 30 |
| bt | Body temperature | Celsius (°C), at the time of ED visit | Continuous | 0 (0) | 36.4 | 35.5 | 38 |
| Wbc | White blood cell count | ×10^3^/µl, initial CBC | Continuous | 142 (2.9%) | 7.1 | 2.9 | 17.9 |
| hb | Hemoglobin level | g/dl, initial CBC | Continuous | 132 (2.7%) | 13.4 | 7.5 | 17.5 |
| plt | Platelet count | ×10^3^/µl, initial CBC | Continuous | 142 (2.9%) | 225 | 64 | 428 |
| bili | Total bilirubin level | mg/dL, initial comprehensive metabolic panel | Continuous | 458 (9.2%) | 0.6 | 0.2 | 2.4 |
| got | Serum aspartate transaminase level | IU/L, initial comprehensive metabolic panel | Continuous | 139 (2.8%) | 22 | 11 | 172 |
| gpt | Serum alanine aminotransferase level | IU/L, initial comprehensive metabolic panel | Continuous | 139 (2.8%) | 20 | 6 | 140 |
| alp | Serum alkaline phosphatase level | IU/L, initial comprehensive metabolic panel | Continuous | 140 (2.8%) | 64 | 32 | 184 |
| prot | Serum total protein | g/dL, initial comprehensive metabolic panel | Continuous | 141 (2.8%) | 7.2 | 5.7 | 8.4 |
| alb | Serum albumin level | g/dL, initial comprehensive metabolic panel | Continuous | 142 (2.8%) | 4.2 | 3.0 | 5.0 |
| bun | Blood urea nitrogen level | mg/dL, initial comprehensive metabolic panel | Continuous | 137 (2.8%) | 15 | 6 | 64 |
| cr | Serum creatinine level | mg/dL, initial comprehensive metabolic panel | Continuous | 143 (2.9%) | 0.88 | 0.49 | 8.23 |
| sodium | Serum sodium level | mmol/L, initial comprehensive metabolic panel | Continuous | 124 (2.5%) | 140 | 125 | 145 |
| potassium | Serum potassium level | mmol/L, initial comprehensive metabolic panel | Continuous | 126 (2.5%) | 4.2 | 3.2 | 5.8 |
| cl | Serum chloride level | mmol/L, initial comprehensive metabolic panel | Continuous | 131 (2.6%) | 104 | 89 | 111 |
| co2 | Total carbon dioxide level | mmol/L, initial comprehensive metabolic panel | Continuous | 835 (16.8%) | 24 | 16 | 31 |
| ca | Serum calcium level | mg/dL, initial comprehensive metabolic panel | Continuous | 127 (2.6%) | 9.2 | 1.1 | 10.4 |
| glu | Serum glucose level | mg/dL, initial comprehensive metabolic panel | Continuous | 125 (2.5%) | 115 | 77 | 357 |

^*^Values of median, 1st percentile and 99th percentile of continuous variables were extracted from the training cohort. All continuous variables were preprocessed for extreme values by replacing 1st and 99th percentiles.

ED, emergency department; N/A, not applicable; CBC, complete blood count.
