# Supplementary material for: Emergency department routine data and the diagnosis of acute ischemic heart disease in patients with atypical chest pain
Source: PLoS One. 2020 Nov 5;15(11):e0241920. doi: 10.1371/journal.pone.0241920 (PMC7644067; doi:10.1371/journal.pone.0241920)
Supplement: S2 Table — Variable importance was determined based on the z value (logistic regression) and gain (XGB) obtained from the training cohort. XGB, extreme gradient boosting; EMS, emergency medical service. (DOCX) [file pone.0241920.s003.docx]

**S2 Table. Full ranking of important variables for the prediction models.**

| Variables |  | Logistic regression rank | Z-value | XGB rank | Gain |
| --- | --- | --- | --- | --- | --- |
| Baseline characteristics | |  |  |  |  |
|  | Age | 1 | 9.08 | 1 | 0.182 |
|  | Sex | 3 | 6.17 | 6 | 0.068 |
|  | EMS use | 22 | 0.22 | 25 | 0.003 |
| Vital signs |  |  |  |  |  |
|  | Systolic blood pressure | 24 | 0.06 | 10 | 0.033 |
|  | Diastolic blood pressure | 16 | 1.62 | 13 | 0.027 |
|  | Heart rate | 5 | -4.76 | 4 | 0.075 |
|  | Respiratory rate | 21 | 0.27 | 24 | 0.004 |
|  | Body temperature | 7 | -3.8 | 9 | 0.042 |
| Laboratory data | |  |  |  |  |
|  | White blood cell | 8 | 3.8 | 3 | 0.081 |
|  | Hemoglobin | 6 | 3.97 | 7 | 0.058 |
|  | Platelet | 17 | 1.49 | 16 | 0.019 |
|  | Total bilirubin | 14 | -1.7 | 22 | 0.007 |
|  | Serum aspartate transaminase | 4 | 5.31 | 5 | 0.074 |
|  | Serum alanine aminotransferase | 9 | -3.35 | 14 | 0.026 |
|  | Alkaline phosphatase | 11 | -2.25 | 12 | 0.028 |
|  | Serum total protein | 18 | -1.46 | 18 | 0.018 |
|  | Serum albumin | 25 | -0.03 | 19 | 0.011 |
|  | Blood urea nitrogen | 13 | -1.71 | 20 | 0.010 |
|  | Serum creatinine | 10 | 3.23 | 11 | 0.032 |
|  | Serum sodium | 15 | 1.7 | 8 | 0.043 |
|  | Serum potassium | 19 | -0.69 | 17 | 0.018 |
|  | Serum chloride | 23 | 0.11 | 21 | 0.007 |
|  | Total carbon dioxide | 20 | -0.67 | 23 | 0.007 |
|  | Serum calcium | 12 | 2.24 | 15 | 0.020 |
|  | Serum glucose | 2 | 6.17 | 2 | 0.108 |

Variable importance was determined based on the z value (logistic regression) and gain (XGB) obtained from the training cohort.

XGB, extreme gradient boosting; EMS, emergency medical service.
